# Real-Life Efficacy of Palbociclib and Ribociclib in Advanced Breast Cancer

**DOI:** 10.3390/curroncol32030161

**Published:** 2025-03-12

**Authors:** Tugay Avci, Mustafa Sahbazlar, Ferhat Ekinci, Atike Pinar Erdogan

**Affiliations:** Department of Medical Oncology, Faculty of Medicine, Manisa Celal Bayar University, Manisa 45030, Turkey; mustafa.sahbazlar@cbu.edu.tr (M.S.); ekinci.ferhat@cbu.edu.tr (F.E.); atike.erdogan@cbu.edu.tr (A.P.E.)

**Keywords:** metastatic breast cancer, palbociclib, ribociclib, CDK 4/6 inhibitors, fulvestrant, letrozol

## Abstract

**Background:** Clinical trials in metastatic hormone receptor-positive (HR+) human epidermal growth factor receptor-2 (HER2)-negative patients have shown that cyclin-dependent kinase 4/6 (CDK 4/6) inhibitors both increase response rates and provide survival benefits. The efficacy of these therapies needs to be supported by real-life data. In this study, we aimed to evaluate treatment response, survival and affecting factors in patients with HR+/HER2− metastatic breast cancer (MBC) who were followed up with CDK 4/6 inhibitors in our center. **Materials and methods:** A retrospective analysis of 120 patients with HR+/HER2− MBC treated with ribociclib or palbociclib in combination with letrozole or fulvestrant was performed. **Results:** Median progression-free survival (mPFS) was 24 months in the general population, 27 months in the ribociclib arm and 20 months in the palbociclib arm, with no significant difference in progression-free survival (PFS) in both arms (*p* = 0.25). The mPFS was longer in the ribociclib + letrozole arm compared to palbociclib + letrozole (27 vs. 20 months, respectively). PFS was also longer in patients receiving ribociclib + fulvestrant compared to palbociclib + fulvestrant but not statistically significant (33 vs. 21 months, respectively). Median overall survival (mOS) was not reached, but 3-year overall survival (OS) was statistically significantly longer in the ribociclib arm (87% vs. 55.5%, respectively, *p* = 0.03). **Conclusion**: Palbociclib and ribociclib are first-line treatment options for metastatic HR+/HER2− disease and have similar efficacy. In our study, while the mPFS was not statistically significant in both arms, the 3-year OS rate was higher in the ribociclib arm and statistically significant. Our findings were confirmed in randomized studies comparing both agents head-to-head.

## 1. Introduction

Breast cancer is the most common cancer in women, with more than 2 million diagnosed each year [1]. Approximately 5% to 10% of patients are metastatic at the time of diagnosis, and those diagnosed with early-stage breast cancer may also develop recurrent advanced or metastatic disease [2]. Although survival is better in early stages, survival in advanced stages is below 30% [3]. Breast cancer is a heterogeneous cancer, and patients are stratified according to the estrogen and progesterone receptor status of the tumor and HER2 status. Treatment plans are made according to the positivity of these markers and prognosis varies according to these subtypes. Considering all breast cancer patients, HR+ breast cancer accounts for 65% of all patients [4]. Although endocrine therapy is the primary treatment in HR+ patients, it has been shown that an increased expression of cyclin-dependent kinase 4 (CDK4) may cause CDK 4/6 inhibitor resistance [5]. With new treatment options introduced in the last 10 years, in patients previously treated only with endocrine therapy, response rates have increased and survival has been prolonged even in an advanced stage. The most important of these therapies are CDK 4/6 inhibitors, which are used in HR+/HER2− patients in adjuvant or metastatic lines.

Uncontrolled cell proliferation is the main mechanism in malignancies and cyclin and CDKs are involved in this control at every stage of the cell cycle. CDK 4/6 inhibitors (palbociclib, ribociclib and abemaciclib) inhibit CDK 4 and 6 in the cell cycle and prevent the transition of the tumor cell from the G1 to S phase. Thus, cancer cell proliferation is prevented. Clinical trials have shown that these drugs are effective in first- and second-line treatment. The MONARCH 3, MONALEESA-2 and PALOMA-2 studies represent pivotal phase 3 studies establishing the role of CDK4/6 inhibitors in the first-line treatment of HR+/HER2− advanced breast cancer [6,7,8]. Each study demonstrated the significant clinical benefit of adding a CDK4/6 inhibitor to endocrine therapy compared to endocrine therapy alone. While all three trials consistently showed a significant improvement in PFS, subtle differences in OS and safety profiles were observed. Ribociclib has shown the most robust OS advantage in phase 3 trials, setting it apart as a leading CDK4/6 inhibitor in terms of long-term survival outcomes.

In terms of second-line efficacy, the MONALEESA-3 and PALOMA-3 studies both evaluated the combination of CDK4/6 inhibitors with fulvestrant in HR+/HER2− advanced breast cancer patients but differed in terms of patient populations and outcomes [9,10]. In MONALEESA-3, both treatment-naive and previously treated postmenopausal patients were included, whereas in PALOMA-3, only previously treated (second-line) pre- or postmenopausal patients were included. Both trials confirmed the benefit of CDK4/6 inhibitors combined with fulvestrant in advanced breast cancer. However, ribociclib (MONALEESA-3) demonstrated a significant improvement in both PFS and OS, positioning it as a more robust option in this setting compared to palbociclib (PALOMA-3), which primarily showed a PFS benefit without a significant OS gain.

As with any treatment, the efficacy of these therapies needs to be supported by real-life data. In our study, we aimed to evaluate the treatment response, survival and factors affecting this in patients with HR+/HER2− MBC who were followed up with CDK 4/6 inhibitors in our center.

## 2. Materials and Methods

This retrospective study enrolled patients aged 18 years and older who visited the Medical Oncology outpatient clinic at Manisa Celal Bayar University Hafsa Sultan Hospital between October 2017 and September 2024 and underwent a minimum of four cycles of treatment with either palbociclib or ribociclib. Patient demographic data, along with clinical and treatment-related characteristics, were collected from medical records.

OS was defined as the duration from the date of diagnosis to death from any cause. PFS was measured as the time from the initiation of treatment to the first documented disease progression or the date of the visit where progression was identified. Additionally, treatment response rates—including the clinical benefit rate (CBR) and objective response rate (ORR)—were assessed, along with factors influencing survival outcomes.

18-Fluoro-deoxyglucose positron emission tomography (FDG-PET)was used for staging and response evaluation. At week 24, patients were evaluated as progressive disease (PD), stable disease (SD), partial response (PR) and complete response (CR) according to the anatomic and metabolic response status on PET-CT.

The statistical analysis was performed using Statistical Package for the Social Sciences (SPSS) 15.0 for Windows. Descriptive statistics for categorical variables were presented as counts and percentages, while numerical variables were summarized using the median, minimum and maximum values. A chi-square test was used to compare proportions across groups. For comparisons of numerical variables between groups, the Student’s *t*-test was applied when the data met the normality assumption, and the Mann–Whitney U test was used when this assumption was not satisfied. Survival rates were assessed using Kaplan–Meier analysis, and risk factors were evaluated through Cox regression analysis. A significance level of 0.05 was set for all statistical tests.

## 3. Results

A total of 120 patients were included in this study. The median age at diagnosis was 53.5 (±11.9) years and the median age at initiation of CDK 4/6 treatment was 58.5 (±11.1) years. A total of 27.5% (n = 33) of the patients were over 65 years of age. A total of 37.5% (n = 45) of the patients received ribociclib and 62.5% (n = 75) received palbociclib. The median age at diagnosis and treatment was statistically significantly higher in patients receiving palbociclib than in patients receiving ribociclib (Table 1). Other patient characteristics were similar in the two arms. In total, 57 (47.5%) of the patients had comorbidities. Hypertension (HT) was the most common comorbidity and was present in 37.5% of patients. Mastectomy was present in 73 patients (recurrent disease) and 47 patients were de novo metastatic. Bone metastases were the most common metastasis and were present in 79.2% of patients. Median CA 15-3 at the beginning of treatment was higher in the palbociclib arm than in the ribociclib arm, but there was no statistically significant difference (42 vs. 25.1, respectively, *p* = 0.29).

The clinical benefit rate was 88.3% in the general population, 95% (43/45) in ribociclib and 84% (63/75) in palbociclib. CR was observed in 4.4% of ribociclib and 2.7% of palbociclib. PR was observed most frequently and was similar in the two groups (55.6% vs. 58.7%). The objective response rates were 60,8% in the general population, 60% in ribociclib and 61.4% in palbociclib.

Progression developed during treatment in 55% of patients. CDK4/6 inhibitors were given as first-line treatment in 35% of patients.

**Figure 1:**
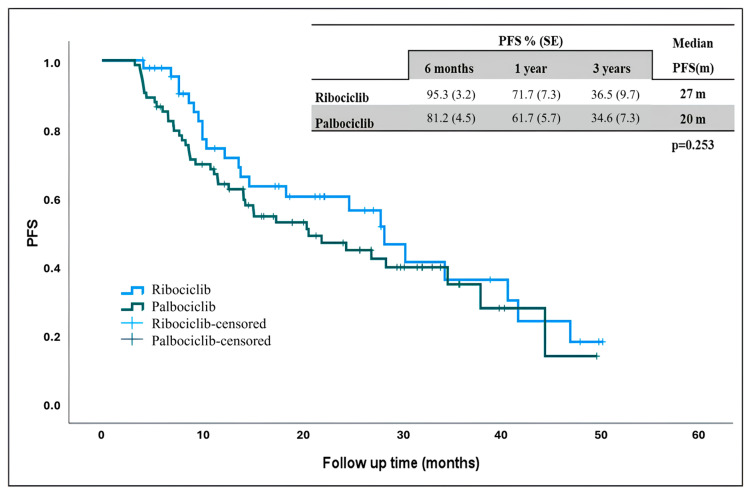
Progression-free survival in patients receiving ribociclib and palbociclib. PFS: progression-free survival, m: months.

The estimated median PFS in the general population was 24 months, 27 months in ribociclib and 20 months palbociclib. Although the median PFS was longer in patients receiving ribociclib, there was no statistically significant difference (Figure 1) (*p* = 0.253).

**Figure 2:**
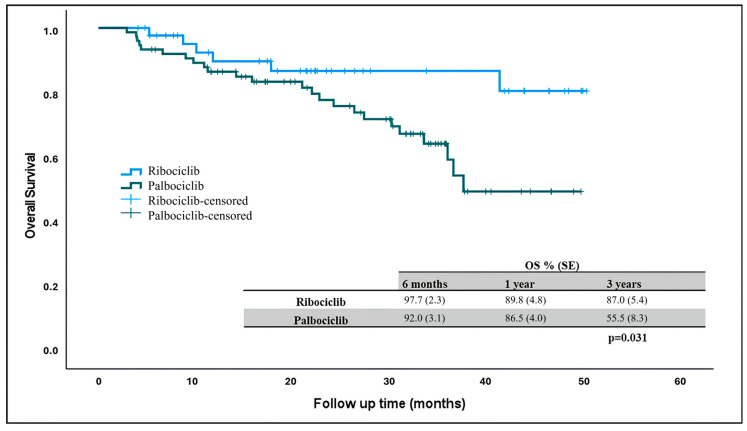
Overall survival in patients receiving ribociclib and palbociclib. OS: overall survival.

At the time of data collection, 90 patients were alive and 6 patients (13%) in the ribociclib arm and 24 patients (32%) in the palbociclib arm had died. The median overall survival was not reached in patients receiving ribociclib and palbociclib, but there was a statistically significant difference in OS rates. Three-year overall survival was higher in patients receiving ribociclib compared to those receiving palbociclib (Figure 2) (*p* = 0.031).

Factors affecting PFS were assessed using a Cox regression analysis (Table 2). While palbociclib and ribociclib show no significant difference in PFS in the univariate analysis (HR: 1.34, 95% CI: 0.80–2.24 and *p*: 0.261), the multivariate analysis suggests a potential trend toward worse outcomes with palbociclib (HR: 1.75, 95% CI: 0.99–3.11 and *p*: 0.053). This warrants further investigation in larger cohorts to clarify the comparative effectiveness of these agents.

Patients with SD have significantly worse PFS compared to those with PR (HR: 3.36, 95% CI: 1.88–6.01 and *p*: < 0.001), highlighting the importance of achieving at least a partial response for better outcomes. CR, however, does not appear to confer additional benefits over PR. Regarding the use of CDK 4/6 inhibitors, a univariate analysis showed that use in later lines increases the risk of progression or death by 20% (HR: 1.2, 95% CI: 1.04–1.39 and *p*: 0.011). A multivariate analysis showed that use in later lines may reduce the risk by 26% and may have a protective effect (HR: 0.74, 95% CI: 0.59–0.93 and *p*: 0.011). This difference may be due to confounding factors such as previous treatments or disease characteristics.

Each additional treatment line was associated with a 28% increased risk of progression or death in the univariate analysis (HR:1.28, 95% CI: 1.16–1.41 and *p* < 0.001). In multivariate analysis (HR: 1.52, 95% CI: 1.28–1.81 and *p* < 0.001), the number of treatment lines had a stronger negative effect on PFS. The risk of disease progression was significantly higher in patients who had received prior multi-line therapy.

When the factors affecting overall survival were evaluated, in the univariate model, there was no significant association between age and overall survival (Table 3). After adjustment, the OR increased to 2.07 (95% CI: 0.59–7.24), indicating a trend toward worse survival in younger patients, but the result was not statistically significant (*p*: 0.253). The wide confidence interval indicates considerable uncertainty in the estimate. Although not statistically significant, the multivariate analysis showed a potential trend toward worse survival in patients with a higher BMI (OR: 2.37, 95% CI: 0.83–6.72 and *p* < 0.105). DM was not significant in the univariate model but became protective in the multivariate model (OR: 0.14, 95% CI: 0.04–0.56 and *p* = 0.005). CAD was significantly associated with worse survival in both models (univariate OR: 1.62 and *p* = 0.016; multivariate OR: 6.12 and *p* = 0.012). HT was not statistically significant in either model. Postmenopausal status was associated with significantly worse survival outcomes in both univariate (OR: 2.22 and *p* = 0.019) and multivariate models (OR: 5.80, 95% CI: 1.34–25.05 and *p* = 0.011), suggesting that postmenopausal women have an elevated risk of death compared to premenopausal women.

ER expression was not significant. PR expression was protective in the univariate model (*p* = 0.021) but lost significance in the multivariate model. Ki67 demonstrated a borderline association with OS in the multivariate model (OR: 1.03 and *p* = 0.051), indicating that higher proliferative activity may be associated with worse survival.

The line of therapy in which CDK4/6 inhibitors were administered showed a significant impact on OS in the multivariate model (OR: 2.09, 95% CI: 1.11–3.92 and *p* = 0.022), indicating that later-line use of CDK4/6 inhibitors was associated with worse survival outcomes. The number of prior treatment lines demonstrated a non-significant trend toward worse survival in the multivariate model (OR: 1.19, 95% CI: 0.63–1.04 and *p* = 0.070). Patients treated with ribociclib exhibited significantly better OS compared to those receiving palbociclib in both univariate (OR: 2.61 and *p* = 0.038) and multivariate models (OR: 3.39, 95% CI: 1.14–10.07 and *p* = 0.028). This suggested potential differences in the efficacy or safety profiles of these agents

The combination of letrozole with CDK4/6 inhibitors was associated with significantly worse OS compared to fulvestrant-based combinations in both univariate (OR: 1.57 and *p* = 0.043) and multivariate models (OR: 2.64, 95% CI: 1.03–6.77 and *p* = 0.043).

## 4. Discussion

Although CDK 4/6 inhibitors are recommended in international guidelines for first-line treatment in patients with hormone-positive HER2-negative metastatic breast cancer, 42 out of 120 patients (35%) received CDK 4/6 inhibitors in the first-line setting in our study. Ribociclib and palbociclib were included in the reimbursement in Turkey on 9 May 2020. Therefore, this treatment could be given to metastatic hormone-positive breast cancer patients who progressed after this date. Therefore, these patients were able to access this treatment in later lines. Although most of the patients received this treatment in the second line and beyond, the clinical benefit rate was 88.3% and the objective response rate was 60% in the whole population.

In the MONALEESA 2 study, the objective response rate in 334 patients who received ribociclib–letrozole was 42.5%, while this rate was 54.5% in those with measurable disease, and the clinical benefit rate was 80.1%. In the PALOMA 2 study, the objective response rate in 444 patients who received palbociclib–letrozole was 42.1%, while this rate was 55.3% in those with measurable disease, and the clinical benefit rate was 84.9% [6,8]. In our study, the objective response rates were 62.5% in the palbociclib + letrozole arm and 64.2% in the ribociclib + letrozole arm, while the clinical benefit rates were 83.3% in the palbociclib + letrozole arm and 96.3% in the ribociclib + letrozole arm. Compared to these studies, the clinical benefit rate of the ribociclib + letrozole treatment and the objective response rates of palbociclib and ribociclib in combination with letrozole were higher in our study. While the median age of patients receiving treatment in these two clinical trials was 62 years, this may have been due to the fact that the patients in our study were younger. Another factor may have been the fact that treatment response evaluation was performed with CT/MR in these clinical trials, whereas it was performed with PET-CT in our study. The fact that our study was retrospective and included patients who received at least four cycles of treatment with CDK 4/6 inhibitors may have led to a bias in data transfer, as patients who developed rapid progression after several cycles of treatment and could not be evaluated for their response may not have been included in our study.

In the MONALEESA-3 study, the objective response rate was 40.9% and the clinical benefit rate was 70.2% in 484 patients who received ribociclib–fulvestrant in the second line, and in the PALOMA 3 study, the objective response rate was 19% and the clinical benefit rate was 66% in 347 patients who received palbociclib–fulvestrant [9,10]. In our study, the objective response rate was 52.9% and the clinical benefit rate was 94.1% in the ribociclib–fulvestrant arm, while the objective response rate was 59.3% and the clinical benefit rate was 85.2% in the palbociclib–fulvestrant arm. In our study, response rates were higher with these treatments compared to clinical trials. Although the majority of the patients in our study consisted of patients receiving second-line and subsequent CDK4/6 treatment, the high response rates may have been due to the relatively lower number of patients receiving fulvestrant and the fact that patients who developed early progression and did not complete four cycles of treatment may not have been included in this study.

Studies have shown that CDK4/6 inhibitors prolong PFS compared with the letrozole treatment alone in metastatic first-line treatment [6,8]. In the MONALEESA 2 trial, the median PFS was 25.3 months in the ribociclib + letrozole arm and 16 months in patients receiving letrozole alone, while in the PALOMA 2 trial, the median PFS was 24.8 months in the palbociclib + letrozole arm and 14.5 months in patients receiving letrozole alone. CDK4/6 inhibitors in combination with fulvestrant have also been shown to prolong PFS compared to fulvestrant alone [9,10]. In the PALOMA 3 study, the mPFS was 11.2 months in patients receiving palbociclib + fulvestrant compared to 4.6 months in the fulvestrant alone arm, and in the MONALEESA 3 study, the mPFS was 20.5 months in patients receiving ribociclib + fulvestrant compared to 12.8 months in patients receiving fulvestrant alone. In our study, the mPFS was 27 months in the ribociclib + letrozole arm, 33 months in the ribociclib + fulvestrant arm, 20 months in the palbociclib–letrozole arm and 21 months in the palbociclib–fulvestrant arm. There was no statistically significant difference in the mPFS between these groups.

There are no phase 3 prospective studies comparing the palbociclib and ribociclib treatments head-to-head in the literature. Looking at real-life data in the literature, in a Danish study of 2069 patients, the mPFS was 42.4 months in the ribociclib arm and 32 months in the palbociclib arm in patients who received CDK4/6 therapy combined with aromatase inhibitors or fulvestrant in the first-line setting, while the mPFS was 18.5 months in the ribociclib arm and 13.6 months in the palbociclib arm in patients who received CDK4/6 in the second-line setting [11]. It is seen that progression-free survival is better when these therapies are given in the first-line setting.

Other retrospective studies conducted in Spain and Qatar found no significant PFS difference between the two agents [12,13]. Again, in a study conducted by the Turkish Oncology Group, the mPFS was similar in the ribociclib and palbociclib arms in patients using CDK4/6 + fulvestrant (12.9 vs. 12.2 months, respectively, *p* = 0.7) [14]. Similar to the literature, when the ribociclib and palbociclib treatment was compared in our study, the mPFS was higher in the ribociclib arm, but not statistically significant (27 months vs. 20 months, respectively, *p* = 0.25).

Overall survival was the secondary endpoints of the PALOMA-1, PALOMA-2 and PALOMA-3 studies, and although there was a prolongation in overall survival in the PALOMA-1 (37.5 vs. 34.5 months; HR, 0.90), PALOMA-2 (53.9 vs. 51.2 months; HR, 0.96) and PALOMA-3 (34.9 vs. 28.0 months; HR, 0.81) studies, no statistically significant difference was found in those receiving the palbociclib treatment compared to the placebo arm [6,9,15]. In contrast to the palbociclib treatment, patients receiving the ribociclib treatment had a statistically significant overall survival advantage compared with placebo in the MONALEESA-2 (63.9 vs. 51.4 months and HR, 0.76) and MONALEESA-3 (67.6 vs. 51.8 months and HR, 0.67) trials [8,10]. However, the treatment arms in the PALOMA-2 trial included patients with an unknown survival status (13.3% vs. 21.2%, respectively). Therefore, data were pooled and a survival analysis was performed again, and the mPFS was 53.8 months in the palbociclib arm and 49.8 months in the placebo arm (HR, 0.92 *p* = 0.21). However, there was still no statistical survival advantage. In terms of real-life data, there are no prospective studies comparing the two agents head-to-head, as in PFS. In a multicenter retrospective study conducted in Germany, 384 patients receiving palbociclib and 231 patients receiving ribociclib were included in the study, and the IPTW-adjusted median OS was 36.7 months in the palbociclib arm and 36.6 months in the ribociclib arm (HR, 0.95) [16]. Again, in multicenter retrospective studies conducted in Portugal and Qatar, no OS difference was found between both agents [12,17].

Although the median OS was not reached in our study, the 3-year overall survival was statistically significantly higher in the ribociclib arm than in the palbociclib arm. The small number of patients receiving ribociclib and the fact that the patients receiving palbociclib were from an older population may have led to this result. Again, when the patients were compared in terms of combined treatments, the 3-year overall survival was higher in patients receiving ribociclib + letrozole than in patients receiving palbociclib + letrozole. However, it was not statistically significant. Again, in a multicenter retrospective study conducted in Turkey, no statistically significant difference was found in 3-year OS rates in patients receiving ribociclib–letrozole and palbociclib–letrozole (74% vs. 61%, respectively) [18]. Regarding CDK 4/6, a fulvestrant combination study involving 522 patients from Turkey found that the estimated OS was 48.5 months in the ribociclib arm and 43.3 months in the palbociclib arm. Although the mOS was numerically higher in the ribociclib arm, statistical significance was not found (*p* = 0.56) [14]. In our study, although 3-year survival was higher in the ribociclib + fulvestrant arm, statistical significance was not found (*p* = 0.15).

The fact that our study was retrospective, that only patients who could receive four cycles of treatment were included in the study and that the number of patients receiving ribociclib was lower than palbociclib can be considered as limitations of this study.

CDK4/6 inhibitors have revolutionized the treatment of HR-positive, HER2-negative breast cancer and show promise for expanding beyond this indication. Ongoing research is investigating their potential in cancers like lung cancer, pancreatic cancer and melanoma, where they could offer new therapeutic options. Combining CDK4/6 inhibitors with immunotherapy, PI3K inhibitors or other agents is a promising strategy to overcome resistance and improve efficacy. These combinations are being actively explored in clinical trials. However, resistance to CDK4/6 inhibitors remains a challenge. Research is focused on understanding the molecular mechanisms behind resistance and developing strategies to overcome it. Additionally, managing side effects such as neutropenia and liver enzyme elevations is crucial to improving patient outcomes.

While progression-free survival (PFS) has improved with these drugs, long-term survival benefits remain under investigation. Extended follow-up studies will provide a clearer picture of their impact on OS. Finally, as we move toward personalized medicine, identifying biomarkers that predict which patients will benefit most from CDK4/6 inhibitors will help optimize treatment strategies and improve outcomes.

## 5. Conclusions

CDK4/6 inhibitors prolonged response rates and survival in metastatic hormone-positive HER2-negative patients. In our study, the mPFS was higher in the ribociclib arm in patients receiving the ribociclib and palbociclib treatment, but not statistically significant. Overall survival was statistically significantly higher in patients receiving ribociclib compared to palbociclib. Prospective randomized studies comparing the two treatments head-to-head involving more patients are needed.

## Figures and Tables

**Table 1:** Demographic and disease characteristics of the whole study group and ribociclib palbociclib groups.

		Total	Ribociclib45 (37.5)	Palbociclib75 (62.5)	
		n (%)	n (%)	n (%)	*p*
Endocrine therapy	Letrozole	76 (63.3)	28 (62.2)	48 (64)	0.845 ^#^
	Fulvestrant	44 (36.7)	17 (37.8)	27 (36)	
Age at diagnosis med. ± SDe		53.5 ± 11.9	48 ± 11.7	57 ± 11.6	0.008 *
Age at treatment med. ± SDe		58.5 ± 11.1	52 ± 11.1	60 ± 10.8	0.038 *
Age at treatment	<65	87 (72.5)	37 (82)	50 (66.6)	0.065 ^#^
	≥65	33 (27.5)	8 (18)	25 (33.4)	
BMI	<25	33 (27.5)	13 (28.9)	20 (26.7)	0.792 ^#^
	≥25	87 (72.5)	32 (71.1)	55 (73.3)	
DM		29 (24.2)	9 (20)	20 (26.7)	0.409 ^#^
CAD		14 (11.7)	3 (6.7)	11 (14.7)	0.186 ^#^
HT		45 (37.5)	13 (28.9)	32 (42.7)	0.131 ^#^
Premenopausal		25 (20.8)	13 (28.9)	12 (16)	0.092 ^#^
Postmenopausal		95 (79.2)	32 (71.1)	63 (84)	
ER% Med.		80	80	80	0.608 **
PR% Med.		2	10	1	0.458 **
Ki 67 Med.		30	30	30	0.828 **
CA 15-3 Med.		30	25.1	42	0.293 **
Luminal	Luminal A	42 (35.0)	17 (37.8)	25 (33.3)	0.621 ^#^
	Luminal B	78 (65.0)	28 (62.2)	50 (66.7)	
Histologic subtype	Ductal	107 (89.2)	40 (88.9)	67 (89.3)	0.465 ^#^
	Lobular	8 (6.7)	2 (4.4)	6 (8)	
	Other	5 (4.2)	3 (6.7)	2 (2.7)	
Recurrent disease		73 (60.8)	31 (68.9)	42 (56)	0.161 ^#^
De novo metastatic		47 (39.2)	14 (31.1)	33 (44)	
Liver metastasis		42 (35)	19 (42.2)	23 (30.7)	0.199 ^#^
Lung metastasis		47 (39.2)	21 (46.7)	26 (34.7)	0.192^#^
Bone metastasis		95 (79.2)	33 (73.3)	62 (82.7)	0.223 ^#^
Brain metastasis		7 (5.8)	3 (6.7)	4 (5.3)	1.000 ^#^
Lymph node metastasis		61 (50.8)	21 (46.7)	40 (53.3)	0.479 ^#^
ECOG PS	0	87 (72.5)	35 (77.8)	52 (69.3)	0.727 ^#^
	1	30 (25)	9 (20)	21 (28)	
	2	3 (2.5)	1 (2.2)	2 (2.7)	
Exitus		30 (25.0)	6 (13.3)	24 (32.0)	0.022 ^#^
Best response to treatment	PR	69 (57.5)	25 (55.6)	44 (58.7)	0.131 ^#^
	SD	33 (27.5)	16 (35.6)	17 (22.7)	
	CR	4 (3.3)	2 (4.4)	2 (2.7)	
	PD	14 (11.7)	2 (4.4)	12 (16)	
	CBR	106 (88.3)	43 (95)	63 (84)	
Progression		66 (55)	23 (51.1)	43 (57.3)	0.507 ^#^
On which line is ciclib used	1.	42 (35)	13 (28.9)	29 (38.7)	0.261 ^#^
	2.	33 (27.5)	10 (22.2)	23 (30.7)	
	3.	27 (22.5)	13 (28.9)	14 (18.7)	
	≥4.	18 (15)	9 (20)	9 (12)	
Number of treatment lines Med.		3	3	2	0.224 **
Follow-up time (months) Med. (min–max)		65 (4–171)	72 (4–147)	57 (4–171)	0.129 **

^#^ Chi-square test, * Student’s *t*-test, ** Mann–Whitney U test, Med: median, BMI: body mass index, DM: diabetes mellitus, CAD: coronary artery disease, HT: hypertension, SDe: standard deviation, PR: partial response, SD: stable disease, CR: complete response, PD: progressive disease, CBR: clinical benefit rate.

**Table 2:** Factors affecting progression-free survival—Cox regression analysis.

	Univariate				Multivariate			
	*p*	HR	95% CI		*p*	HR	95% CI	
Palbociclib vs. Ribociclib	0.261	1.34	0.80	2.24	0.053	1.75	0.99	3.11
Combination agent (Ref:letrozole) Fulvestrant	0.987	1.00	0.60	1.66	0.942	0.98	0.57	1.67
Best response to treatment (Ref:PR)	<0.001				<0.001			
SD	<0.001	3.46	1.98	6.05	<0.001	3.36	1.88	6.01
CR	0.968				0.969			
PD	<0.001	13.6	6.63	28.1	-			
On which line is ciclib used	0.011	1.20	1.04	1.39	0.012	0.74	0.59	0.93
Number of treatment lines	<0.001	1.28	1.16	1.41	<0.001	1.52	1.28	1.81

PR: partial response, SD: stable disease, CR: complete response, PD: progressive disease, HR: hazard ratio.

**Table 3:** Risk factors affecting overall survival—Cox regression analysis.

		Univariate				Multivariate			
		*p*	OR	95% CI		*p*	OR	95% CI	
Age Category	<65	0.415	0.72	0.33	1.58	0.253	2.07	0.59	7.24
	≥65								
BMI	≥25	0.762	0.88	0.39	1.99	0.105	2.37	0.83	6.72
	<25								
DM		0.486	0.73	0.30	1.78	0.005	0.14	0.04	0.56
CAD		0.329	1.62	0.62	4.24	0.016	6.12	1.40	26.70
HT		0.390	1.39	0.66	2.92	0.180	0.32	0.06	1.70
Menopause status	(premenopausal)	0.140	2.22	0.77	6.38	0.019	5.80	1.34	25.05
	postmenopausal								
Percent ER		0.533	1.01	0.99	1.02	0.150	1.02	0.99	1.04
Percent PR		0.029	0.98	0.97	1.00	0.021	0.98	0.96	1.00
Ki67		0.087	1.02	1.00	1.03	0.051	1.03	1.00	1.05
Luminal A vs. Luminal B		0.808	0.91	0.42	1.95	0.420	1.74	0.45	6.63
Histological subtype	(Ref: Ductal)	0.144				0.092			
	Lobular	0.214	2.15	0.64	7.23	0.099	3.60	0.79	16.51
	Other	0.097	3.52	0.80	15.56	0.085	5.21	0.80	34.10
Recurrent disease		0.882	0.95	0.45	1.99	0.848	1.10	0.43	2.79
On which line is ciclib used		0.290	1.11	0.91	1.36	0.022	2.09	1.11	3.92
Number of treatment lines		0.840	1.02	0.87	1.19	0.070	0.63	0.39	1.04
Palbociclib vs. Ribociclib		0.038	2.61	1.05	6.46	0.028	3.39	1.14	10.07
Combination agent (Ref: Fulvestrant) Letrozole		0.274	1.57	0.70	3.53	0.043	2.64	1.03	6.77

OR: Odds ratio, BMI: body mass index, DM: diabetes mellitus, CAD: coronary artery disease, HT: hypertension, ER: estrogen receptor, PR: progesterone receptor.

## Data Availability

The raw data supporting the conclusions of this article will be made available by the authors upon request.
